# Current and Future Burden of Prostate Cancer in Songkhla, Thailand: Analysis of Incidence and Mortality Trends From 1990 to 2030

**DOI:** 10.1200/JGO.17.00128

**Published:** 2018-01-30

**Authors:** Christian S. Alvarez, Shama Virani, Rafael Meza, Laura S. Rozek, Hutcha Sriplung, Alison M. Mondul

**Affiliations:** **Christian S. Alvarez**, **Shama Virani**, **Rafael Meza**, **Laura S. Rozek**, and **Alison M. Mondul**, University of Michigan School of Public Health, Ann Arbor, MI; and **Shama Virani** and **Hutcha Sriplung**, Prince of Songkla University, Songkhla, Thailand.

## Abstract

**Purpose:**

Prostate cancer is the second most common malignancy among men worldwide, and it poses a significant public health burden that has traditionally been limited mostly to developed countries. However, the burden of the disease is expected to increase, affecting developing countries, including Thailand. We undertook an analysis to investigate current and future trends of prostate cancer in the province of Songkhla, Thailand, using data from the Songkhla Cancer Registry from 1990 to 2013.

**Methods:**

Joinpoint regression analysis was used to examine trends in age-adjusted incidence and mortality rates of prostate cancer and provide estimated annual percent change (EAPC) with 95% CIs. Age-period-cohort (APC) models were used to assess the effect of age, calendar year, and birth cohort on incidence and mortality rates. Three different methods (Joinpoint, Nordpred, and APC) were used to project trends from 2013 to 2030.

**Results:**

Eight hundred fifty-five cases of prostate cancer were diagnosed from 1990 to 2013 in Songkhla, Thailand. The incidence rates of prostate cancer significantly increased since 1990 at an EAPC of 4.8% (95% CI, 3.6% to 5.9%). Similarly, mortality rates increased at an EAPC of 5.3% (95% CI, 3.4% to 7.2%). The APC models suggest that birth cohort is the most important factor driving the increased incidence and mortality rates of prostate cancer. Future incidence and mortality of prostate cancer are projected to continue to increase, doubling the rates observed in 2013 by 2030.

**Conclusion:**

It is critical to allocate resources to provide care for the men who will be affected by this increase in prostate cancer incidence in Songkhla, Thailand, and to design context-appropriate interventions to prevent its increasing burden.

## INTRODUCTION

In 2012, prostate cancer was the second most commonly diagnosed cancer and the fifth leading cause of cancer death among men worldwide.^1,2^ Prostate cancer incidence varies up to 25-fold across world regions, with the highest age-standardized rates (ASRs) in Western developed countries, such as the United States.^1^ However, prostate cancer mortality varies less across regions (approximately 10-fold) than incidence rates, with the highest age-standardized mortality rates (ASMRs) estimated from less developed regions such as sub-Saharan Africa and the Caribbean.^1,3^ Accounting for growth and aging of the world population, the global burden of prostate cancer is expected to increase to 1.7 million new cases and nearly half a million deaths by 2030.^1,4^

In Asia, reported incidence rates of prostate cancer are much lower than most Western developed countries.^3-8^ However, over the past decade, prostate cancer incidence rates have increased rapidly in several Asian populations.^4-6,8-10^ For instance, the incidence rates in East Asia increased on average 7.2% per year from 2005 to 2009.^5^ Similarly, mortality rates increased in some Asian countries, ranging from 5.3% per year in Shanghai, China (from 1985 to 2009), to 13.4% per year in South Korea (from 1985 to 2002).^6,9^ The rapid increase in the burden of prostate cancer in Asia may be partly a result of an aging population and adoption of Westernized lifestyles as a consequence of economic development.^5,6,8,9^

In Thailand, the nationwide incidence rates of prostate cancer have increased at an average annual percent change of 2.7% over the past two decades.^2^ The mean annual ASR increased from 4.9 prostate cancer cases per 100,000 person-years in 1995 to 1999 to 7.1 prostate cancer cases per 100,000 person-years in 2010 to 2012.^11,12^ However, reports from the Thai National Cancer Institute show regional differences in the incidence of prostate cancer, with a higher incidence rate in southern Thailand compared with the northeast region (ASR, 10.4 *v* 4.1 prostate cancer cases per 100,000 person-years, respectively).^12^ Southern Thailand is a unique region as a result of its ethnic and cultural composition, where approximately 30% of the population is Muslim, mostly of Thai ethnicity.^13^ It is clear that there is a need to comprehensively assess cancer incidence and mortality by region in Thailand, rather than just at the national level; to our knowledge, this has not been done, particularly in southern Thailand. We undertook an analysis investigating trends in the incidence and mortality of prostate cancer using data from the Songkhla Cancer Registry in southern Thailand from 1990 to 2013 and projecting prostate cancer rates to 2030.

## METHODS

### Study Population

**Fig 1:**
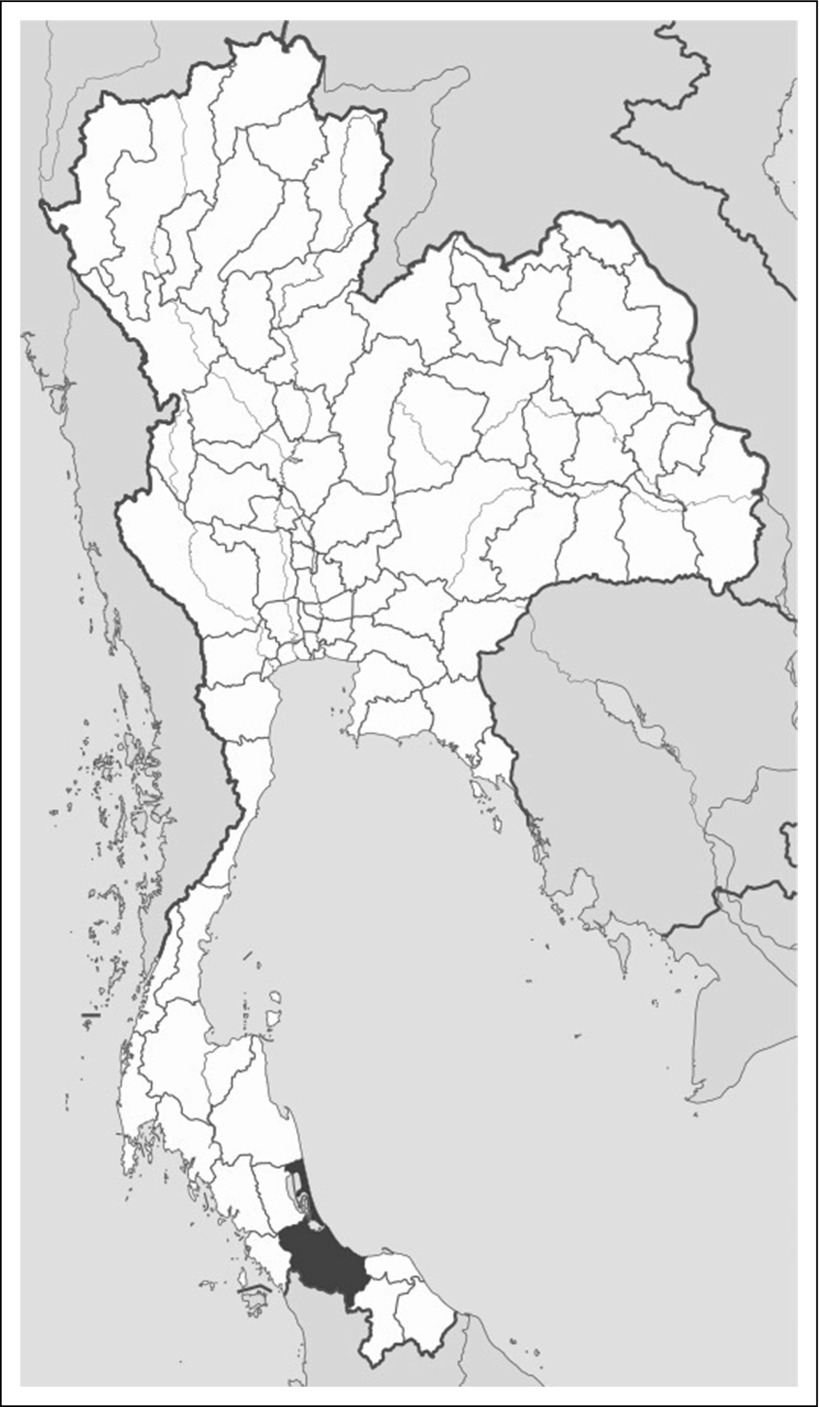
Map of Thailand (Songkhla province is shaded).

Songkhla is a southern province of Thailand, located on the eastern side of the Malay Peninsula (Fig 1). In 2010, the population of Songkhla was approximately 1.5 million, of which 48.8% were male.^13,14^ Estimates from the Thai National Statistical Office show that 25% of the population in the Songkhla province is Muslim and 75% is Buddhist.^14,15^ Furthermore, approximately 15% of the population in Thailand is older than age 60 years, with a life expectancy of 72 years for men.^16,17^

### Data Source

Data on incident prostate cancers were obtained from the Songkhla Cancer Registry from 1989 to 2013. This registry has been described in detail previously.^13,18-20^ Briefly, the Songkhla Cancer Registry was established in 1989 and covers 16 districts in the province of Songkhla.^13^ It actively captures cases of cancer from 23 sources, including the three tertiary referral hospitals for cancer in the province (Songklanagarind Hospital, Hat Yai Hospital, and Songkhla Hospital); community, private, and special hospitals; and the provincial health and population registration office.^13,15,18-20^ Cancer case data are mainly collected from hospital and pathology records with the highest standard of quality, using strict protocols for cancer case identification.^13^ According to the cancer report in Thailand (volume VII, 2007 to 2009), 87% of prostate cancers in the Songkhla Cancer Registry were histologically verified, and only 2.4% were obtained from death certificates.^21^ Completeness is > 95%, evaluated by capture-recapture methods.^22^ This registry has been included in the International Agency for Research on Cancer’s publication *Cancer Incidence in Five Continents* since volume VIII (1993 to 1996).^18^

### Data Extraction and Variables

Cancer cases were extracted using the International Classification of Diseases, 10th revision, code for malignant neoplasm of the prostate (C61). Complete information on cancer cases was available from 1990 to 2013 (n = 855). Variables in the registry included dates of diagnosis, last contact, and death; vital status; tumor grade, stage, and extent; age at diagnosis; religion; and district of residence.

Population denominators were obtained from decennial census data in 1990, 2000, and 2010 conducted by the Thai National Statistical Office. The annual intercensal population structure in Songkhla was estimated by 5-year sex-specific age groups, using a log-linear function between consecutive censuses. The population beyond 2010 was estimated by the Office of the National Economic and Social Development Board.^15,19,20^

### Statistical Analysis

Descriptive statistics (medians and percentages) were generated for the variables in the cancer registry. Age-specific incidence and mortality rates of prostate cancer were calculated for 24 calendar periods between 1990 and 2013 (1-year intervals) and 18 different 5-year age groups and adjusted to the world standard Segi population.^23^ Incidence and mortality rates used for comparison purposes in this study were also adjusted to the world standard Segi population.

### Analysis of Incidence and Mortality Trends

Joinpoint regression analysis was conducted to examine trends in ASRs and ASMRs for prostate cancer using the Joinpoint Regression Program version 4.2.0.2 (https://surveillance.cancer.gov/joinpoint/). Joinpoint regression identifies statistically significant trend change points (joinpoints) and the rate of change (estimated annual percent change [EAPC]) in each trend segment using a Monte Carlo permutation method.^13,19^ A maximum number of four joinpoints was allowed in the analysis to best describe the trend of the data. We also used age-period-cohort (APC) models to assess the effects of age, calendar year, and birth cohort on the prostate cancer risk and mortality (Data Supplement, Methods).^13^

### Prediction of Prostate Cancer Incidence and Mortality

To project the incidence and mortality rates of prostate cancer in Songkhla, Thailand, through 2030, three independent models were used to compare the results across these methods; these were Joinpoint, Nordpred, and APC model projections as performed by Virani et al^13^ in her analysis of breast cancer in Songkhla, Thailand. Ninety-five percent prediction intervals [PIs] and validation were conducted for the Joinpoint model only. Details of these methods are described in the Data Supplement.

## RESULTS

**Fig 2:**
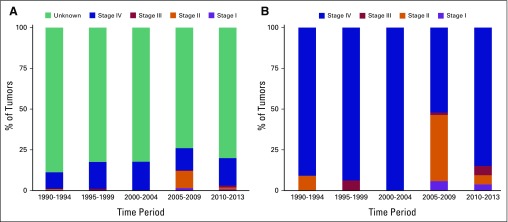
Prostate cancer stage distribution across 5-year periods for (A) all tumors (N = 855) and (B) tumors that were staged only (n = 175).

Eight hundred fifty-five cases with prostate cancer were diagnosed from 1990 to 2013. The median age at diagnosis was 74 years (quartile 1 to quartile 3, 67 to 80 years). The majority of prostate cancer cases were Buddhist (89.6%) and the rest Muslim. Most of the prostate cancer cases were unstaged (79.8%); among those who were staged, 3.5%, 17.9%, 2.9%, and 75.7% had stage I, II, III, and IV disease, respectively. We observed a statistically significant change in the stage distribution over time (*P* < .001, Data Supplement). This change was largely a result of the proportion of unstaged tumors decreasing with a concomitant increase in stage II tumors during 2005 to 2009 (Figs 2A and 2B and Data Supplement).

### Joinpoint

**Fig 3:**
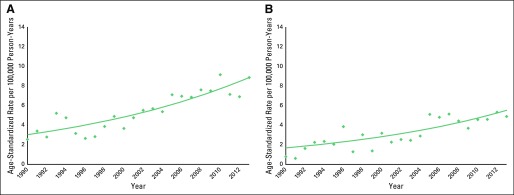
Age-adjusted (A) incidence and (B) mortality rates of prostate cancer in Songkhla, Thailand, from 1990 to 2013 for men all ages by Joinpoint analysis. Estimated annual percent change was 4.8% (95% CI, 3.6% to 5.9%; *P* < .05) for incidence and 5.3% (95% CI, 3.4% to 7.2%; *P* < .05) for mortality. The points show the observed rates, and the lines indicate the incidence and mortality trends.

Prostate cancer incidence rates in Songkhla increased significantly from 1990 to 2013 at an EAPC of 4.8% (95% CI, 3.6% to 5.9%; *P* < .05; Fig 3A). The ASR increased approximately three-fold, from 2.55 to 8.87 prostate cancer cases per 100,000 person-years in 1990 and 2013, respectively. Similarly, the mortality rate of prostate cancer in Songkhla increased significantly since 1990 at an EAPC of 5.3% (95% CI, 3.4% to 7.2%; *P* < .05; Fig 3B). The ASMR increased nearly six-fold, from 0.80 to 4.93 deaths per 100,000 person-years in 1990 and 2013, respectively. In a sensitivity analysis excluding the first 2 years of data, the mortality EAPC was similar (EAPC, 4.72%; 95% CI, 2.9% to 6.6%; *P* < .05). Thus, subsequent mortality analyses did not exclude these data.

### APC

**Fig 4:**
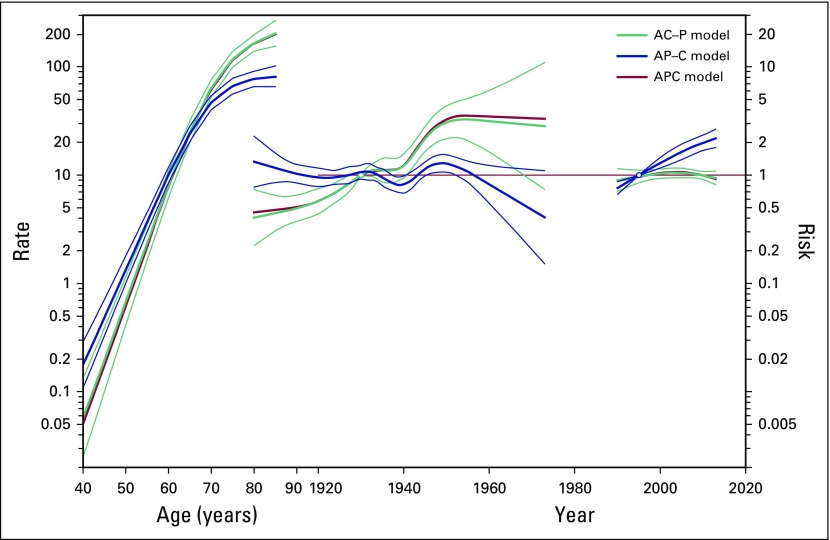
Age-period-cohort trend analysis for incidence of prostate cancer (1990 to 2013) in men of all ages in Songkhla, Thailand. A, age; C, cohort; P, period.

**Fig 5:**
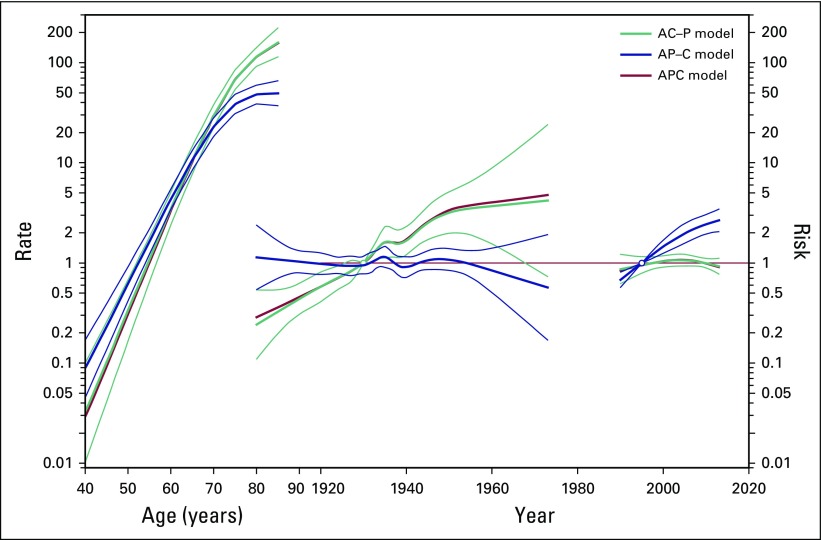
Age-period-cohort trend analysis for mortality of prostate cancer (1990 to 2013) in men of all ages in Songkhla, Thailand. A, age; C, cohort; P, period.

Figure 4 shows the APC incidence trend analysis for each of the models (APC, AC-P [age-cohort model], and AP-C [age-period model]). The incidence trends in the models show that the incidence rates of prostate cancer increase exponentially (linear in log-scale) with age (Fig 4, left). We observed that younger cohorts have a higher risk of prostate cancer (Fig 4, center) and that the risk of prostate cancer increases with calendar year (Fig 4, right). The risk is approximately 2 times higher (95% CI, 1.68 to 2.34) in 2010 versus 1995. The APC analysis for mortality yielded similar results for all models (Fig 5). The age-cohort model provides the best fit for the data in both incidence and mortality APC trend analysis, and the greatest difference of deviance residual is observed after cohort is removed from the full APC model, suggesting that birth cohort is the most important factor driving the increased incidence and mortality rates of prostate cancer (Table 1).

**Table 1:**
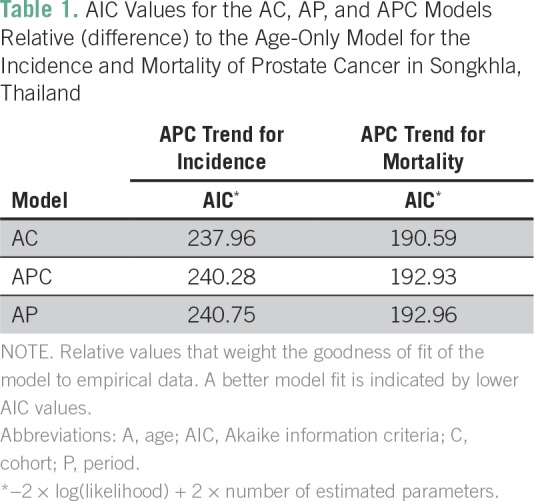
AIC Values for the AC, AP, and APC Models Relative (difference) to the Age-Only Model for the Incidence and Mortality of Prostate Cancer in Songkhla, Thailand

### Projections

**Fig 6:**
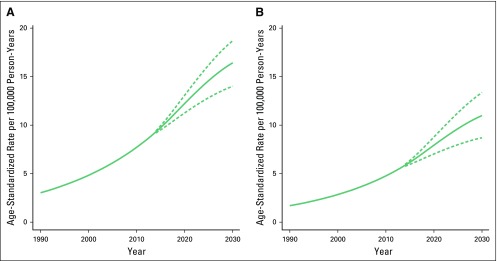
Prostate cancer (A) incidence and (B) mortality trend projections to 2030. Joinpoint method with 95% prediction intervals (PIs). The continuous lines are the projected incidence and mortality trends, and the dashed lines show the 95% PIs.

Prostate cancer incidence and mortality are estimated to continue increasing in the next decade (Fig 6). By 2030, incidence rates are expected to double from the 2013 rates, increasing from 8.9 to 16.4 prostate cancer cases per 100,000 person-years (95% PI, 14.0 to 18.7 prostate cancer cases; Fig 6A). Incidence projections were similar using APC and Nordpred (Data Supplement). By 2030, mortality rates will increase from those observed in 2013, from approximately five to 11 deaths per 100,000 person-years (95% PI, 8.7 to 13.4 deaths). Mortality projections were similar across the methods used (Data Supplement).

Results for incidence and mortality projections were validated using data from 2006 to 2010 to project rates for 2011 to 2013. The projected data for 2011 to 2013 closely matched the observed data for both incidence and mortality (Data Supplement).

## DISCUSSION

This first in-depth look at the trends of prostate cancer in Songkhla, Thailand, demonstrates that there has been a significant increase in prostate cancer incidence and mortality since 1990 (Figs 3A and 3B) likely as a result of changes in sociodemographic and lifestyle factors of the Thai population. In addition, the burden of prostate cancer is expected to continue to increase through 2030 (Fig 6).

The increasing trends in prostate cancer in Songkhla are similar to those observed in other areas of Thailand as well as across Asia. In Chiang Mai (northern Thailand), the incidence and mortality rates of prostate cancer increased at an EAPC of 3.3% (95% CI, 2.2% to 4.4%) from 1983 to 2009 and 2.7% (95% CI, -4.4% to 10.4%) from 1980 to 1994, respectively.^6^ Similarly, in Shanghai, China, the prostate cancer incidence and mortality EAPCs were 3.2% (95% CI, 0.3% to 6.8%) from 1991 to 2004, and 5.3% (95% CI, 4.7% to 6.0%) after 1985, respectively.^9^ Other Asian countries have reported similar results.^6^ However, in the United States, the incidence and mortality of prostate cancer have decreased at a rate of 1.1% (95% CI, 0.4% to 1.8%) and 3.4% (95% CI, 3.3% to 3.6%) from 1990 to 2013, respectively.

Although prostate cancer incidence rates are increasing in Thailand, the rates remain low compared with developed Western countries.^6^ In 2013, the ASR of prostate cancer in the United States was 74.8 prostate cancer cases per 100,000 person-years, approximately nine-fold higher than the rate in Songkhla, Thailand, in the same year (ASR, 8.87 prostate cancer cases per 100,000 person-years).^24^ This difference in incidence rates between the United States and Thailand is partially explained by the use of prostate-specific antigen (PSA) for prostate cancer screening in the United States.^25,26^ However, the use of PSA screening does not completely explain these differences because rates in Western countries that do not routinely do population-based PSA screening, such as the United Kingdom, are still substantially higher than those in Thailand (ASR, 73.2 prostate cancer cases per 100,000 person-years).^1^ There are no official guidelines on population-based screening for prostate cancer in Thailand or any other Asian countries, except for Japan, where screening rates remain low (12.2% in 2011).^27^

Asian men may also be at a reduced genetic risk of prostate cancer. Asian Americans have lower prostate cancer rates compared with white Americans (37.2 and 69.0 prostate cancer cases per 100,000 person-years, respectively).^28^ Furthermore, genetic studies on prostate cancer have observed substantial racial differences between white and Asian populations. Importantly, the *TMPRSS2*-*ERG* fusion, which is associated with poorer prognosis,^29^ is more prevalent in whites (approximately 50%) than Asian populations (8% to 21%).^5,29^

Prostate cancer incidence and mortality rates in Songkhla increased at approximately the same EAPC during the study period. It should be noted that the increase in mortality over time occurred despite a slight downward shift in the stage distribution at diagnosis in later periods. The mortality-to-incidence ratio (MIR) of prostate cancer is remarkably higher in Songkhla (MIR, 0.56) compared with the United States (MIR, 0.09), even though the difference in prostate cancer mortality rates is currently small (US and Songkhla mortality rates, 8.5 and 5.57 deaths per 100,000 person-years, respectively). However, if prostate cancer mortality rates remain stable in the United States, the projected mortality rate in Songkhla will surpass the US rate by 2030 (10.99 *v* 8.5 deaths per 100,000 person-years, respectively). The higher MIR in Songkhla is partially a result of the large proportion of prostate cancer tumors diagnosed at advanced stages. Our study found that 75.7% of staged tumors were diagnosed at an advanced stage versus only 4% of tumors diagnosed in the United States.^30^ PSA screening contributes to diagnosing patients at early stages in the United States; however, PSA screening remains controversial, and the benefits of early detection must be weighed against the risk of overtreatment, adverse effects, and impaired quality of life.^31-33^ Nonetheless, even in Western countries where population-wide PSA screening is not conducted, the stage distribution is still much lower than in Thailand (eg, 17% of patients diagnosed at advanced stage in United Kingdom).^34^ Designing interventions to diagnose prostate cancer at earlier stages in Thailand will be instrumental in reducing prostate cancer mortality in this population.

The adoption of a more Western lifestyle, particularly a poorer diet and less physical activity, has been speculated to increase the incidence of cancer in this region. This is supported by the increase in rates observed by birth cohort in the APC analyses and the cohort effects from the AC-P (age-cohort) model. Thailand has undergone both social and economic transitions over the past three decades that have shifted dietary patterns toward a diet high in fat, meat, and total energy intake as well as lowered physical activity.^35^ Furthermore, studies have suggested that environmental factors may play a role in the risk of progression of prostate cancer to adverse outcomes.^36^ In fact, several risk factors (eg, higher body mass index, smoking, reduced lycopene intake) have been observed for lethal or aggressive prostate cancer, but not for indolent disease.^36^ Because more patients in Songkhla are diagnosed with advanced-stage disease, it is likely that etiologic factors in this population are similar to those identified for aggressive or lethal prostate cancer in the United States.

We also considered whether introduction of universal health coverage by the Thai National Health Security Office in 2002 may have contributed to the increase in incidence and mortality. However, we observed a linear increase in both incidence and mortality over time that did not differ between the periods before and after introduction of universal health care. In addition, the stage distribution at diagnosis remained similar before and after this introduction. If improved access to health care was strongly influencing rates, we would expect to see an increase or no change in incidence with a stage shift toward lower stages at diagnosis and, perhaps, reduced mortality as a result of improvements in treatment. Thus, the pattern we observed is not consistent with the introduction of universal health coverage having a strong influence on prostate cancer incidence or mortality. We also considered whether awareness of prostate cancer as a possible diagnosis by health care providers may have increased in recent decades, potentially contributing to increased trends. However, again, we did not observe any substantial downshift in the staging of prostate cancer at diagnosis as we might expect under this scenario. Further research is necessary to address these hypotheses.

This study was, to our knowledge, the first to explore the current and future trends of prostate cancer in Songkhla. Each of the methods we used for the projection analysis (Joinpoint, Nordpred, and APC) has different limitations, including the assumption of a Poisson distribution for the method presented in the main findings (ie, the Joinpoint method). However, our results were essentially the same no matter which model was used, indicating the robustness of our findings. Our data come from a population-based cancer registry, which allows us to extrapolate the results to the entire province of Songkhla; in addition, the data have been collected with the highest standard of quality to obtain accurate estimates.^13^ Nonetheless, it is difficult to estimate the number of cancer cases not captured by the registry in the province of Songkhla. Although universal health care has been available since 2002, some individuals residing in rural villages may not choose to access health care services and may prefer to use traditional medicine.^13^

Another limitation of this study is that mortality rates represent all-cause mortality (not prostate cancer–specific mortality), which might have led to prostate cancer mortality estimates that were slightly too high. However, it should be noted that the resulting rates are similar to those estimated in other studies in Asia, suggesting that our results are reasonably accurate.^9^ An alternative strategy for identifying deaths would have been to use data from death certificates from the Thai Ministry of Health. However, death certificate data in Thailand is relatively poor quality with considerable misclassification.^37^ A study conducted in 2003 found that the agreement between cause of death recorded in hospital records and that from death certificates was only 25%.^37^ Had we used death information from these records instead, we likely would have substantially underestimated the prostate cancer mortality rate in this population.

In conclusion, prostate cancer incidence and mortality have increased in Songkhla, Thailand, since 1990 and are expected to continue to increase through 2030. Lifestyle changes may be the most important factors driving the increased incidence and mortality of prostate cancer in Songkhla. Additional studies should evaluate the role of the improvement in access to health care as well as awareness of prostate cancer in Thailand. It is critical to allocate resources to provide care for men who will be affected by the increased burden of disease in this population. In addition, further research is important to identify strategies for the control of prostate cancer in Songkhla, Thailand, including the impact of the introduction of screening programs.
